# Global indicators of the environmental impacts of invasive alien species and their information adequacy

**DOI:** 10.1098/rstb.2023.0323

**Published:** 2024-05-27

**Authors:** Marie V. Henriksen, Eduardo Arlé, Arman Pili, David A. Clarke, Emili García-Berthou, Quentin Groom, Bernd Lenzner, Carsten Meyer, Hanno Seebens, Reid Tingley, Marten Winter, Melodie A. McGeoch

**Affiliations:** ^1^ Department of Landscape and Biodiversity, Norwegian Institute of Bioeconomy Research, Trondheim 7031, Norway; ^2^ Macroecology & Society, German Centre for Integrative Biodiversity Research (iDiv), Halle-Jena-Leipzig, Puschstraße 4, 04103 Leipzig, Germany; ^3^ School of Zoology, George S. Wise Faculty of Life Sciences, Tel Aviv University, Tel Aviv 6997712, Israel; ^4^ School of Biological Sciences, Monash University, Clayton 3800, Victoria, Australia; ^5^ Securing Antarctica's Environmental Future, School of Biological Sciences, Monash University, Clayton 3800, Victoria, Australia; ^6^ GRECO, Institute of Aquatic Ecology, University of Girona, 17003 Girona, Spain; ^7^ Meise Botanic Garden, 1860 Meise, Belgium; ^8^ Division of BioInvasions, Global Change & Macroecology, Department of Botany and Biodiversity Research, University of Vienna, Rennweg 14, 1030 Vienna, Austria; ^9^ Institute of Geosciences and Geography, Martin Luther University Halle-Wittenberg, 06099 Halle, Germany; ^10^ Institute of Biology, Leipzig University, 04103 Leipzig, Germany; ^11^ Senckenberg Biodiversity and Climate Research Centre, Senckenberganlage 25, Frankfurt 6325, Germany; ^12^ EnviroDNA Pty Ltd, 95 Albert Street, Brunswick, Victoria 3056, Australia; ^13^ sDiv, Synthesis Centre, German Centre for Integrative Biodiversity Research (iDiv) Halle-Jena-Leipzig, Puschstraße 4, 04103 Leipzig, Germany

**Keywords:** biological invasions, ecological niche modelling, impact evidence, impact mechanisms, indicator uncertainty, knowledge gaps

## Abstract

Monitoring the extent to which invasive alien species (IAS) negatively impact the environment is crucial for understanding and mitigating biological invasions. Indeed, such information is vital for achieving Target 6 of the Kunming–Montreal Global Biodiversity Framework. However, to-date indicators for tracking the environmental impacts of IAS have been either lacking or insufficient. Capitalizing on advances in data availability and impact assessment protocols, we developed environmental impact indicators to track realized and potential impacts of IAS. We also developed an information status indicator to assess the adequacy of the data underlying the impact indicators. We used data on 75 naturalized amphibians from 82 countries to demonstrate the indicators at a global scale. The information status indicator shows variation in the reliability of the data and highlights areas where absence of impact should be interpreted with caution. Impact indicators show that growth in potential impacts are dominated by predatory species, while potential impacts from both predation and disease transmission are distributed worldwide. Using open access data, the indicators are reproducible and adaptable across scales and taxa and can be used to assess global trends and distributions of IAS, assisting authorities in prioritizing control efforts and identifying areas at risk of future invasions.

This article is part of the theme issue ‘Ecological novelty and planetary stewardship: biodiversity dynamics in a transforming biosphere’.

## 1. Introduction

Invasive alien species (IAS) are a major driver of global biodiversity loss [1]. The pressing need to understand and manage this ubiquitous and ongoing environmental threat has long been recognized by the international community [2] and is reflected in a number of policy targets for IAS [3]. Specifically, Target 6 of the Kunming–Montreal Global Biodiversity Framework (K–M GBF) calls parties to ‘eliminate, minimize, reduce and or mitigate the impacts of invasive alien species on biodiversity and ecosystem services' [4]. Information needed to support Target 6, therefore, includes data on species impacts and how these are changing over time, which requires regular and effective impact monitoring and estimation [5]. These data would then populate one or more indicators capable of robustly and meaningfully providing spatially explicit information on change in IAS impacts.

Invasive alien species impacts can be defined as a measurable change to the properties of an ecosystem by an alien species [6]. Associated indicator development for tracking IAS impacts over time has lagged behind other research and policy dimensions of biological invasions [7]. The only established indicator that tracks changes in IAS impacts is the IUCN Red List Index for IAS (RLI) [7], which assesses changes in the extinction risk of those species threatened by IAS [8] and is used at continental (e.g. [9,10]) and global scales [11].

Although the RLI is tractable and relevant for tracking IAS impacts on species extinction risk [12], it is deficient in a number of key components [7]. For example, it does not always provide information on those species causing the impact, the variety of ways in which they bring about an impact (their impact mechanisms, e.g. predation or herbivory), or on the types of impact they inflict (e.g. ecosystem effects or habitat degradation) [13]. Individual IAS commonly have multiple mechanisms and types of impact (e.g. [14,15]), and particular areas are increasingly exposed to impacts from multiple species [16]. It is also crucial that the location and timing of impact events are comprehensively recorded [7]. This information provides the foundation for effectively prioritizing both species and areas for prevention and control. A multi-species and spatially explicit invasive alien species indicator is thus needed that provides decision-makers with information on the degree of impact and the ecological mechanisms through which this impact is realized. Although advancements in quantitative impact assessments have been made [17], semi-quantitative approaches such as the Environmental Impact Classification for Alien Taxa (EICAT; [18]) offer a near-term alternative to delivering impact information and, importantly, have been adopted by the IUCN [19] and proposed for use at national level [20].

A common impediment to indicator development, however, has been the scarcity of available data for quantifying impacts at appropriate spatial and temporal scales [5,21]. Environmental impact evidence for IAS must be taxonomically, spatially and temporally explicit if it is to inform decision-making at relevant scales [7]. Although major strides in information delivery on IAS have been made, key data gaps remain, notably on the number of IAS populations established outside their native range and significant deficits in information on IAS environmental impacts [15,22]. Indeed, a dearth of environmental impact knowledge has constrained the usefulness of using EICAT as an impact indicator across taxa and in many countries (but see [20,23]). For example, there are inadequate data to assess the impacts of two thirds of invasive alien insects [14]. Identifying and addressing information shortfalls are an essential step towards ensuring that IAS monitoring is overseen by robust decision-making [7]. Recent efforts in biodiversity data mobilization and synthesis, such as the Global Register of Introduced and Invasive Species (GRIIS) [24,25], and approaches and tools for data integration [21,26] open new opportunities for developing IAS impact indicators and their automation. They also provide an opportunity for identifying and reporting on strategic gaps in IAS knowledge, supporting Target 21 of the K–M GBF [4].

Here we design and demonstrate indicators that can be used to monitor the multiple environmental impacts of IAS assemblages over time, using the developments outlined above as a foundation. The impact indicators distinguish (i) realized (i.e. observed) from (ii) potential environmental impacts. A complimentary indicator of the adequacy of data underlying the impact indicators, i.e. an IAS information status indicator, is also proposed. This indicator targets key dimensions of the data needed for the impact indicators, and it can be used to guide and track improvements in impact information as well as to interpret the uncertainty associated with the impact indicators. We demonstrate how these indicators may be used at a global scale using invasive alien amphibian species.

## 2. Methods

### (a) Impact indicators

The proposed indicators of IAS environmental impact combine information on the mechanisms and magnitudes of impact from multiple IAS, and capture both spatial and temporal change (table 1). As stated above, for this purpose we distinguish between realized and potential environmental impacts (defined below) and the indicators are developed accordingly. Potential and realized impact indicators are each expressed by sub-indicators (detailed below) that can be conveyed as a time series, measured as the cumulative number of IAS over time (based on first introduction records) and as a map. For both realized and potential impact, the cumulative number of species is chosen as the measure of trends in impact since there is not yet a system in place for regular updates to assessments of impact evidence. Maps are generated from regional lists of IAS and, in the case of potential impact, modelled distributions of IAS within those regions. The indicators are demonstrated using GRIIS regions [25], which are primarily, though not exclusively, delineated by country administrative boundaries (exceptions include islands or exclaves that are classified independently as regions).

**Table 1.** Realized and potential impact sub-indicators including priority species subset considered, interpretation and implications for management. Realized impact sub-indicators (1.1–1.3) are quantified from regional impact evidence, while potential impact sub-indicators (2.1–2.3) are quantified based on global impact evidence. Proposed sub-indicators for which impact data are not currently available are indicated with asterisks.

sub-indicators	priority species subset	indicator interpretation		implications for policy and management
		distribution	trends	
1.1. Realized total impact	All observed IAS with regional impact evidence	Degree of impact documented regionally	Growth in impact of established invaders over time across regions (global) or per region (regional)	To prioritize management of established invaders with confirmed regional impacts
1.2. Realized impact mechanisms*	Observed IAS with regional impact mechanism evidence	Degree to which different impact mechanisms are documented to influence native species assemblages	Growth in the impact of established invaders on native biodiversity and ecosystems via specific impact mechanisms	To prioritize management of established invaders for conservation of native taxa or ecosystems that are vulnerable to specific mechanisms of impact
1.3. Realized worst invaders*	Observed IAS with major or severe regional impact evidence	Degree of impact by the regionally worst invaders documented currently	Growth in impact of the worst established invaders over time	To prioritize management of worst invaders that are known to have the largest negative impact on native species and ecosystems
2.1. Potential total impact	All observed IAS with global impact evidence	Degree of impact from the regional IAS potentially unobserved	Growth in potentially unobserved impact of IAS over time across regions (global) or per region (regional)	To prioritize management of invasive species (including new, unassessed and established invaders) in a region
2.2. Potential impact mechanisms	Observed IAS with global impact mechanism evidence	Degree to which different impact mechanisms could be influencing native species assemblages	Growth in the potential impact of invasive species on native biodiversity and ecosystems via specific impact mechanisms	To prioritize management of invasive species (including new, unassessed and established invaders) for conservation of native taxa or ecosystems that are vulnerable to specific mechanisms of impact
2.3. Potential worst invaders	Observed IAS with major or severe global impact evidence	Degree to which impact could be occurring regionally due to globally important invaders	Growth in the potential impact of the worst invasive species on native biodiversity and ecosystems via specific impact mechanisms	To prioritize management of the worst invaders globally that are most likely to have future negative impacts on native species and ecosystems

‘Realized impact’ is defined as: *the presence of one or more IAS known to negatively affect the environment (biodiversity and ecosystems) in a given region*. Only alien species that are classified as invasive in GRIIS [24,25], and that have been assessed for their environmental impacts in the region, are included.

‘Potential impact’ is defined as: *the presence of one or more IAS known to negatively affect the environment (biodiversity and ecosystems) in any location globally*. This includes alien species that are classified as invasive in GRIIS [24,25] and with impacts (including mechanisms and magnitude) that have been assessed at a global, but not regional, level. Sub-indicators of ‘potential impact’ (see §2a(i)) thus rely on the assumption that alien species with a history of impact elsewhere are likely to become impactful if introduced in other regions [27,28], and by extension, likely to have the same mechanisms and magnitude of impact. The ‘potential impact’ sub-indicators are visualized as a modelled distribution of impact across regions where impact has the potential to occur, expressed as summed relative likelihood of occurrence of the IAS across their current invaded range (spatial index of potential impact; electronic supplementary material, S1). This results in a predicted distribution of IAS assemblages within impacted regions.

#### (i) Sub-indicators

**Figure 1.**
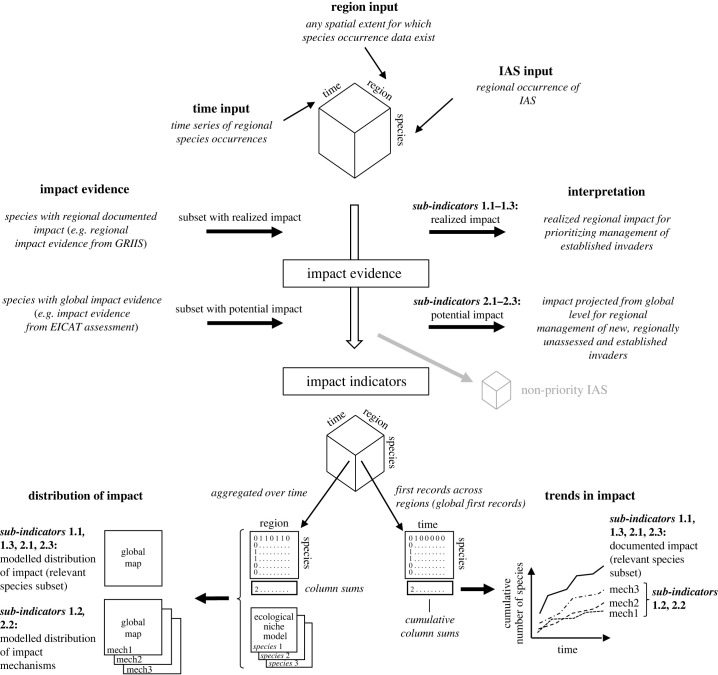
Generating indicators of invasive alien species impact including realised and potential impact indicators of regional invasive alien species assemblages. Each sub-indicator is illustrated as both spatial distribution of impact and trends in impact over time. Impact evidence used includes total number of species with documented impact, degree of impact severity and mechanisms of impact. In the case of potential impact, distribution is modelled based on ecological niche models within regions where species in the relevant subset occur (according to GRIIS). The scale at which impact evidence is documented (e.g. global or regional) changes the relevance and interpretation of indicators for management of IAS assemblages.

Both realized and potential impact are each expressed as a set of three sub-indicators, i.e. total impact, impact mechanisms and worst invaders (table 1), conveying complementary information on three priority species subsets, respectively: (1) the total number of IAS with documented impact (regardless of impact mechanism and magnitude) as a representation of the overall degree of IAS impacts; (2) the number of species for each impact mechanism (regardless of impact magnitude), which measures the vulnerability of environments to specific impact mechanisms; and (3) the number of worst invaders, i.e. species assessed to impact native systems at a community level (regardless of impact mechanism), denoting the impacts from the most impactful IAS only (in EICAT this includes species ‘major’ and ‘massive’ impacts; [18]). Sub-indicators are numbered as 1.1–1.3 for realized impact and 2.1–2.3 for potential impacts in table 1 and figure 1. Notably, since realized and potential indicators differ in the spatial extent at which impact evidence is gathered (regionally for realized impact sub-indicators and globally for potential impact sub-indicators), their interpretation and relevance for policy also differ (table 1).

#### (ii) Assessing uncertainty

Measuring the uncertainty of information conveyed by indicators is crucial for their relevance and scientific validity [7]. Any impact indicator will be biased by insufficient data on regional occurrences of alien species and their impacts, which are both notoriously difficult to estimate and generally subject to time lags (e.g. [29,30]), but which can be improved using modelling approaches [5,21]. The approach also rests on key assumptions for species distribution modelling (e.g. niche conservatism; unbiased sampling effort in environmental space; equilibrium with the environment, which is notoriously violated for alien species; and that sums of relative likelihoods of occurrence approximate species richness [31,32] - electronic supplementary material, S1). The impact indicators should therefore be considered alongside the information status indicator (below), which is proposed to assess the uncertainties of IAS information used in the impact indicators and the consequences for their interpretation.

### (b) Information status indicator

An ‘information status’ indicator informs on the status and availability of the empirical data on the introduction, range dynamics and impacts of IAS, with the purpose of supporting the interpretation of the impact indicators. It indicates growth in the coverage of the introduction history in specific regions, spatiotemporally-explicit occurrences and impact data for IAS, respectively. This indicator can be disaggregated by taxonomic group, region or country and is composed of three sub-indicators based on three key dimensions of IAS information [33]: 1) Introduction date (*In*): whether there is information on the first introduction of a species in the region where it is listed as alien; 2) Range dynamics (*Rd*): if there is minimum occurrence data adequacy to enable producing range dynamics studies on the invasion status for the species within the region; and 3) Impact (*Im*): the availability of information on the impacts caused by the species in this region. The information status indicator should be interpreted alongside the total number of known alien species of the taxon of interest in a given region. We refer to this as ‘information burden’ to reflect the fact that regions with many introduced species will have a large burden in terms of gathering the information needed to adequately manage and report information status. Readily accessible information on introduction dates and range dynamics, and on the impacts of amphibian IAS both globally and at the regional level, was used to calculate the composite IAS information status indicator and its three sub-indicators (electronic supplementary material, S2, table S8).

### (c) Data requirements

Georeferenced occurrence records, in addition to regional and global impact evidence, are required for indicator calculation. Biases and gaps in biodiversity databases are well recognized, as is the deficit in available impact information. Furthermore, introduction dates are often difficult to ascertain, which also relates to time lags in the reporting of IAS introduction events. Thus, the proposed indicators are developed using currently available data but importantly can be updated regularly in the future as new data become available.

The indicators combine global and regional evidence of impact with types of impact (from GRIIS [24]) and EICAT assessments [34]), regional occurrences of IAS (from GRIIS), modelled species distributions (using ecological niche models to predict the distribution of potential impact) and first records of introduction [35] to quantify the spatial distribution of multiple impacts and track their change over time. Impact indicator development capitalizes on recent efforts to collate impact evidence from regional species lists in the GRIIS database [24,25] and in global EICAT assessments [18]. The realized impact sub-indicators use the regional evidence of impact from GRIIS to assess the impact of regionally established invaders. Data on impact evidence for two of the proposed sub-indicators, 1.2 and 1.3 (realized impact mechanisms and realized worst invaders, respectively) are currently not available because there is not yet an organized effort to produce and publish impact mechanisms and magnitude systematically assessed at a regional (country) level. As such, we omit them from subsequent indicator demonstrations. The potential impact sub-indicators use global impact evidence (available from EICAT assessments) to assess the potential impact of the regional IAS assemblage including new, unassessed and established invaders. Over time, the indicators can be re-calculated with updated versions of GRIIS and new global EICAT assessments.

### (d) Demonstration of indicators

Using the SInAS workflow [26], we integrated data from GRIIS and the first records dataset, thus providing the backbone of country-level locality and introduction event data required for each indicator. The workflow was used to harmonize: i) taxon names according to the backbone taxonomy of the Global Biodiversity Information Facility (GBIF; [36]); ii) the Darwin Core terminology of occurrence status and establishment means [37]; and iii) the first records in countries as single years [35]. Multiple forms of uncertainty are associated with assigning alien and IAS status to populations and species [38]. To ensure that indicators are correctly interpreted and are repeatable, it is essential that they are underpinned by systematic decisions that operationalize the definition of species included and excluded and that specify the data used. From the output of the workflow, amphibian records were used to demonstrate the indicators. This included 75 naturalized amphibians in 82 countries (according to the GRIIS database), of which 11 are priority species (assessed as invasive; [34]). We evaluate the impact and information status indicators against the nine criteria for policy relevance and scientific validity identified by Vicente *et al*. [7].

#### (i) Impact indicators

Occurrence information for each of the 11 priority amphibian species is complemented by the full suite of associated impact types. Each species' global impact potential was mapped based on climatically suitable areas within regions where the species occurs (according to GRIIS), as predicted by ecological niche models (full methods provided in electronic supplementary material, S1). As such, impact potential is synonymous with the ecological niche model's predicted climatic suitability. For a given subset of species, the potential impact is the summed climatic suitabilities for all IAS in the subset (electronic supplementary material, S1).

#### (ii) Information status indicator

To demonstrate the information status indicator, the 75 amphibian species considered naturalized in at least one country were used (i.e. GRIIS region) [25]. Determination of each country's IAS information status first requires calculating each of the three information status sub-indicators.

Each sub-indicator contributes equally to the composition of the information status indicator. (1) Introduction date evidence (*In*)—the percentage of species listed by GRIIS for a given region with an available date of first introduction [35]. (2) Impact evidence (*Im*)—the percentage of species listed by GRIIS for a given region for which the impact has been assessed (i.e. designated as ‘isInvasive’ in GRIIS [24]). (3) Range dynamics evidence (*Rd*)— this sub-indicator relies on the availability of occurrence data published through GBIF [39]. To calculate the *Rd* sub-indicator, each species–region combination was assessed as meeting a minimum criterion of having at least 10 records available for each time slice of 5 years within the 50 years considered (1970–2019). Minimum data adequacy is defined here as having some minimum number of occurrence records per species needed to enable modelling of IAS distributions and their change (i.e. range dynamics) [21]. The minimum number is set low to make this target achievable (given adequate sampling effort), irrespective of the species' rarity or regional population dynamics, and it can be revised as required. All unique records for each species listed, from the period 1970 to 2019, were considered, thus excluding duplicate records with the same coordinates and same date. We calculated the *Rd* sub-indicator for each region as the percentage of species–time slice combinations for which at least 10 records are available. Finally, we calculated an overall measure of regional completeness by averaging the three sub-indicators (electronic supplementary material, S2, table S8).

Our proposed indicators meet eight of the nine criteria for policy relevance and scientific validity [7]. The only criterion not met relates to indicator establishment, which can only be achieved once the indicators are implemented and tested across a range of contexts over time (electronic supplementary material, S2, table S9).

## 3. Results

### (a) Environmental impacts of invasive alien amphibians

**Figure 2.**
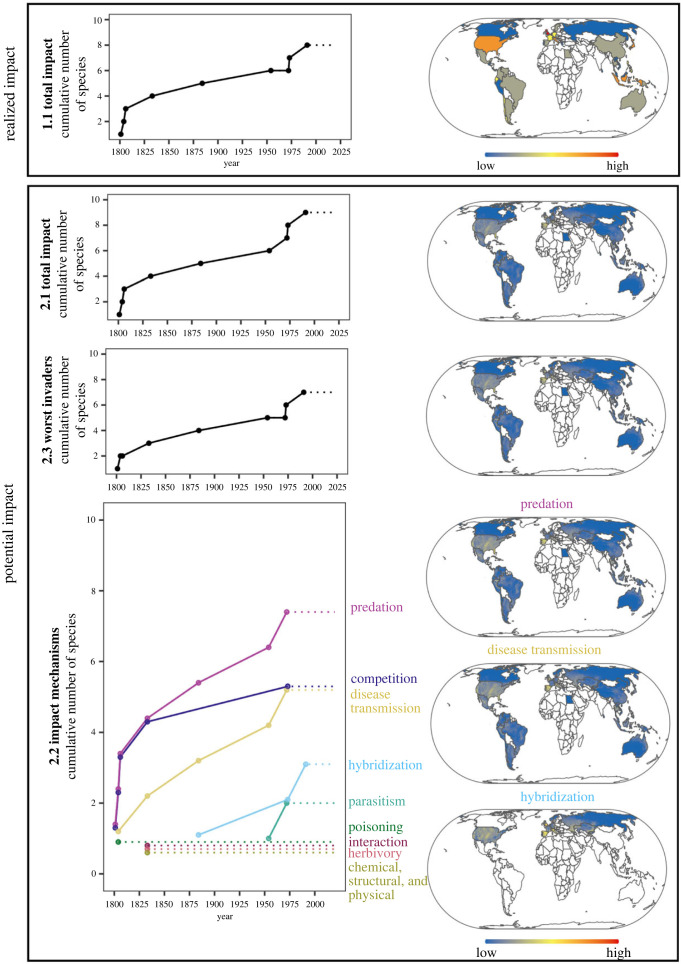
The global growth (left) and distribution of the realized and potential impact to biodiversity associated with amphibian invasions. The current values of the sub-indicators (1.1, 2.1–2.3) are assumed to be at the level of the most recent record of introduction (dashed lines, e.g. 7 for predation). The distribution of potential impact represents the global risk of impact expressed as summed global climate suitability of species across regions and in countries where these multiple species are known to have been introduced, estimated using ecological niche models (electronic supplementary material, S1). In each case, the sums are based on the relevant species and subset of types of impact of concern, among the total of 11 species and 9 impact types (electronic supplementary material, S1).

Trends in the total realized impact of established invaders (sub-indicator 1.1; derived from regional impact evidence) showed that the number of species with realized impact has increased more severely since around the 1970s compared to the previous 100 years (figure 2, 1.1). The distribution of this realised impact is uneven and highest in the UK, followed by the USA, Japan and Indonesia (figure 2, 1.1). Trends in the total and worst potential impacts have been rising steadily since the 1800s, with a marked increase from 1950s–1970s onwards (figure 2, 2.1 and 2.3). The potential impact mechanism sub-indicator (table 1, 2.2), displaying the most detailed level of global impact evidence used, shows that the global increase in potential impact is dominated by predatory amphibians, followed by those that transmit diseases or have a high competitive ability, while comparatively few amphibian IAS express impact through the potential to hybridize (figure 2, 2.2). Potential impacts of invasive alien amphibians via disease transmission are widely distributed globally, whereas hybridization potential occurs predominantly across parts of Europe and Russia. Predation, disease transmission and competition were the most widespread potential impact mechanisms across countries and often also the most common mechanisms within specific countries (figure 2, sub-indicator 2.2). Other mechanisms, such as parasitism, poisoning and interaction with other invasive species were present but less prevalent (few species in few countries; electronic supplementary material, S1 figure S1 and figure S2).

### (b) Information status for invasive alien amphibians

**Figure 3.**
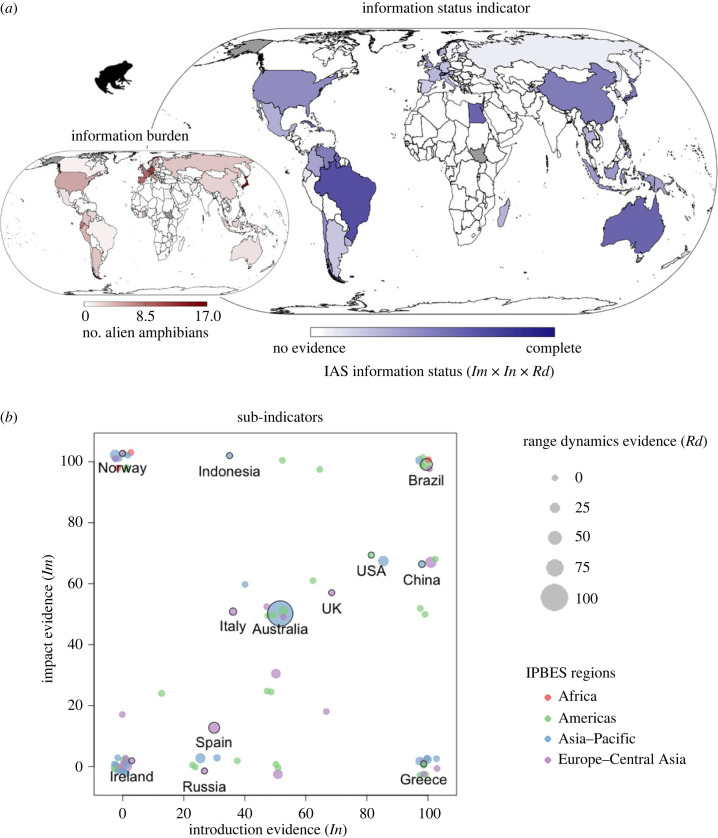
Indicator of the information status of invasive alien species and their impacts. (*a*) Values of the indicator expressed as the average of the three information sub-indicators (shown in panel (*b*)), ranging from 0 (no evidence) to 100% (complete evidence), to be interpreted alongside the inset showing the information burden (number of known alien amphibian species) per country. Countries in white have no reported amphibian IAS, and grey countries have no data to calculate the indicator. (*b*) The completeness of information on invasive alien amphibians disaggregated by sub-indicator: *Im*, percentage of IAS with national-level evidence of impact; *In*, percentage of these species for which introduction date is available; *Rd*, point size represents the percentage of species for which at least 10 occurrence records per five year period (1970–2019) are available. Each point is a country and selected country names are shown as examples and highlighted in black outline. Colours show the IPBES Region of the country.

Impact indicator trends are considered to be relatively reliable for countries with a high information status and, even more so, with a low information burden (figure 3*a*). For amphibians this includes countries such as Brazil and Australia (figure 3*a*). Impact indicator results for countries with low information status—and in particular in combination with high information burden, such as Spain—are uncertain and the apparent absence of impact should be interpreted with caution. Most of Africa, the Arabian Peninsula and the Indian subcontinent have no known invasive amphibians listed (figure 3*a* inset), while Alaska and South Sudan have no data (figure 3*a*). Many countries have complete information for one sub-indicator only, including a cluster of countries with introduction evidence but little to no impact evidence (e.g. Greece), with another cluster (including Norway) having the opposite (figure 3*b*). Importantly, the information status indicator is intended for tracking information change within countries over time, and overall at a global scale. It is not intended for the purpose of comparing the efforts of particular countries, especially given the uneven distribution of the information burden across them (figure 3*a* inset).

From the composite indicators, Brazil is an example of a country with comparatively complete overall evidence on amphibian IAS, including complete information for two of the three sub-indicators (*In* and *Im*; figure 3). At the same time, however, Brazil has a low information burden with just two IAS amphibians (figure 3*a* inset; sub-national species translocations were not included in our workflow [40]). For example, for the American bullfrog (*Lithobates catesbeianus*), which is known to been introduced to Brazil, there is both a date of first record and a verified record of its impact in the country (figure 3*b*, impact evidence and introduction date evidence). However, a minimum adequate number of occurrence records for this species is available for only 20% of the half-decade time slices since its introduction (range dynamics evidence, figure 3*b*), yielding an overall information status indicator score of 77% (figure 3*a*). Brazil does have a second invasive alien amphibian that is not yet included in its GRIIS Country Checklist, as well as four within-country species translocations [40]. Over time, this indicator will bridge the disconnect that exists between research data and data available for national reporting by highlighting information in this way. Furthermore, it could be expanded to encompass sub-national scale resolution. Countries with high information status and low information burden could achieve 100% on each sub-indicator axis with moderate effort. By contrast, the investment needed to achieve full information coverage of the indicator is much higher in Japan, which has the most amphibians listed as IAS [25] (figure 3*a* inset).

## 4. Discussion

The K–M GBF Target 6 calls for countries to eliminate, minimize, reduce and/or mitigate the impacts of invasive alien species on biodiversity and ecosystem services, and to track progress within a Theory of Change framework [4]. Doing so requires information on the outcomes of actions to reduce the threat to biodiversity from invasions, i.e. for Target 6, this would comprise changes over time in invasive alien species impacts. Here we developed and demonstrated indicators of the impact of invasive alien species to identify, characterize and track both the mechanisms and magnitude of these impacts on the environment. Information availability is a key determinant of target tracking and success, not only for invasive alien species, and this is recognized explicitly (as Target 21) in the Kunming–Montreal Global Biodiversity Framework [4]. We also provide a complimentary indicator of information status for the essential information components of the impact indicator in order to identify data gaps and track progress in efforts to fill these. By monitoring the acquisition of data, key gaps can be identified and targeted in future investment.

### (a) Strengths and caveats

Effective indicators for tracking biological invasions should adhere, as far as possible, to a well-established set of criteria for robust indicators [7]. The indicators presented here align with most criteria considered essential for policy relevance and scientific validity. Currently, global impact assessments (e.g. EICAT) are lacking for most taxa and, at the regional level, country-specific assessments of impact mechanisms and magnitudes are not systematically assessed and reported (although see [14]).

The indicators presented here can be expressed at any spatial grain for which species occurrence and impact evidence exist. Here they were demonstrated per country (grain) and at a global extent using the occurrence data of species with impact evidence published in GRIIS [24,25]. The subsets of IAS species used for populating the sub-indicators (all IAS, IAS with specific mechanisms and worst invaders) can also in principle be modified to include any priority species subset of interest. Naturally, these indicators are not yet established (i.e. this is the only criterion from Vicente *et al*. [7] that is not met), but this publication provides a key step in that direction by providing the rationale and methods for a policy-relevant suite of impact indicators quantified from open access data sources.

Key for the uptake of any newly developed indicator is its ease of interpretability. Difficulties interpreting the output of an indicator can at best prevent uptake and at worst lead to inappropriate policies. Although the proposed indicators rely on substantial data collecting efforts and database integration [26], they are themselves derived from relatively simple calculations. The potential impact sub-indicators are valuable for prioritizing and implementing management actions for IAS likely to arrive in a certain region, or for alien species invasive elsewhere for which a regional impact has not been detected or assessed. These indicators can therefore also inform early warning and eradication plans for IAS by highlighting areas of invasion risk. The realized sub-indicators are directly useful as tools for prioritizing management efforts. Moreover, the modular design of the indicators supports their flexible application, enabling both comprehensive assessments for a given region of interest and fair comparisons of IAS impacts across regions based on their common sub-indicators and assessed taxa.

Unless the impact indicators are viewed in the context of the information status indicators, perceptions of IAS impacts will be biased by insufficient data on regional occurrence of IAS and their impacts, both of which are notoriously undersampled, difficult to estimate and generally subject to ecological and publishing time lags [41–43]. Our demonstration case of the indicators for amphibians points to ubiquitous information gaps; no country where amphibians have been introduced shows high completeness in all three information status sub-indicators. Results from the impact indicators should therefore always be considered alongside the information status indicator to assess gaps in information and their consequences for impact interpretation.

### (b) Future developments

Indicators necessarily rely heavily on key data sources (see §2). While these data sources represent recent and significant advances in data collation and accessibility for IAS, they are themselves incomplete and dynamic. A key role of the information status indicator is therefore to illustrate not only the need for new data on IAS to fill gaps and to track the ongoing spread of IAS, but also to highlight the importance of sustained investment in generating, improving and maintaining such data as essential for providing the evidence needed for policy and management for IAS. It is likely that available impact evidence relevant for these indicators will grow, including, for example, EICAT assessments for new taxa (e.g. [14]), regional impact evidence (countries and islands) from GRIIS [24], information on socioeconomic impacts (e.g. [44]) and more sophisticated estimates of compound and synergistic impacts (e.g. using network analysis [45]). However, data initiatives for IAS, such as GRIIS, are to date largely supported by primary research and volunteer efforts (e.g. [24,35]). The mechanism that GRIIS provides for countries to update their IAS checklists is key to sustaining these indicators and improving the reliability of impact indicator results over time. Additionally, given the need for rapid delivery of indicators that are reproducible, comparable and repeatable, these data sources need to be machine readable and interoperable so that the calculation of indicators can ultimately be automated. Furthermore, access to Open Source software to calculate these indicators would both facilitate adoption and ensure transparency [46].

The range dynamics evidence sub-indicator is illustrated here using GBIF as a data source. However, depending on the country for which the user applies the analysis, there may be specific datasets for occurrence data (e.g. SpeciesLink, www.splink.cria.org.br; Atlas of Living Australia, www.ala.org.au). Similarly, thematic datasets may also provide more appropriate information (e.g. Reef Life Survey, reeflifesurvey.com; OBIS, obis.org). Finally, occurrence data are rife with uncertainties and taking steps to filter out the most unreliable records might improve robustness (e.g. [47]). The temporal frame of the range dynamics sub-indicator considers 5-year time steps. This frame could be longer or shorter, depending on the life span of the focal taxon (like generation times; see [48]) or how rapidly it is known to spread.

Although impact is presented as a cumulative number of IAS in the proposed indicators, the indicators could be modified to show the total number of species in a given period of time if regular updates to country checklists are implemented in the future. This would enable the indicators to capture reduction in impact risk over time, with successful local eradication and control efforts resulting in a decline in the number of species associated with a particular type of impact over time, along with a decline in the spatial distribution of the impact (figure 2). Future development of the impact indicators could also include modelled species trends over time to better accommodate bias and add estimates of uncertainty [49] and to provide a more robust assessment of temporal change in impact.

The realized impact indicator includes two sub-indicators for which data do not currently exist (indicators 1.2 and 1.3). These are included to highlight future data needs. While country-level checklists of IAS are being compiled in GRIIS, this does not currently include mechanisms and magnitude of impact. To understand the impact of IAS on native biodiversity and ecosystems and enable prioritization of management measures, this level of detail is needed. Evidence of impact at the regional level is labour-intensive to gather but is currently being compiled by different research groups [25,50].

## 5. Conclusion

As the multiple forms of global change and their impacts on the environment accelerate and compound, it becomes increasingly important to identify the ‘what, where and how’ of IAS impacts to tailor and prioritize appropriate responses. There has been remarkable progress over the past decade regarding information collation and delivery and the biodiversity data standards and workflows needed to deliver biodiversity, and in particular invasion, indicators. The synthetic evidence on the extent and severity of IAS impacts presented in the recent IPBES Assessment of Invasive Alien Species and their control [1] demonstrates the urgency of better understanding and monitoring this threat to biodiversity and ecosystems. It is therefore both feasible and important that by the 2030 deadline for reporting on Target 6 of the Kunming–Montreal Global Biodiversity Framework, information-rich indicators of impact be adopted at country and global scales.

## Data Availability

R code to generate the indicators is available from the GitHub repository: https://github.com/EduardoArle/Impacts [51]. Supplementary material is available online [52].

## References

[1] IPBES. 2023 Thematic Assessment Report on Invasive Alien Species and their Control of the Intergovernmental Science-Policy Platform on Biodiversity and Ecosystem Services (eds HE Roy, A Pauchard, P Stoett, T Renard Truong). Bonn, Germany: IPBES secretariat.

[2] Wilson JRU et al. 2020 Is invasion science moving towards agreed standards? The influence of selected frameworks. NeoBiota **62**, 569-590. (10.3897/neobiota.62.53243)

[3] UNEP. 2010 Decision adopted by the Conference of the Parties to the Convention on Biological Diversity at its tenth meeting. (See https://treaties.un.org/doc/source/docs/UNEP_CBD_COP_DEC_X_1-E.pdf.)

[4] UNEP. 2022 Decision adopted by the Conference of the Parties to the Convention on Biological Diversity. (See https://www.cbd.int/doc/decisions/cop-15/cop-15-dec-04-en.pdf.)

[5] McGeoch M, Jetz W. 2019 Measure and Reduce the Harm Caused by Biological Invasions. One Earth **1**, 171-174. (10.1016/j.oneear.2019.10.003)

[6] Ricciardi A, Hoopes MF, Marchetti MP, Lockwood JL. 2013 Progress toward understanding the ecological impacts of nonnative species. Ecol. Monogr. **83**, 263-282. (10.1890/13-0183.1)

[7] Vicente JR, Vaz AS, Roige M, Winter M, Lenzner B, Clarke DA, McGeoch MA. 2022 Existing indicators do not adequately monitor progress toward meeting invasive alien species targets. Conserv. Lett. **15**, e12918. (10.1111/conl.12918)

[8] Butchart SHM et al. 2010 Global Biodiversity: Indicators of Recent Declines. Science **328**, 1164-1168. (10.1126/science.1187512) 20430971

[9] McGeoch MA, Shaw JD, Terauds A, Lee JE, Chown SL. 2015 Monitoring biological invasion across the broader Antarctic: a baseline and indicator framework. Global Environ. Change **32**, 108-125. (10.1016/j.gloenvcha.2014.12.012)

[10] Rabitsch W, Genovesi P, Scalera R, Biała K, Josefsson M, Essl F. 2016 Developing and testing alien species indicators for Europe. J. Nat. Conserv. **29**, 89-96. (10.1016/j.jnc.2015.12.001)

[11] McGeoch MA, Butchart SHM, Spear D, Marais E, Kleynhans EJ, Symes A, Chanson J, Hoffmann M. 2010 Global indicators of biological invasion: species numbers, biodiversity impact and policy responses: Invasive alien species indicator: 2010 Biodiversity Target. Div. Distrib. **16**, 95-108. (10.1111/j.1472-4642.2009.00633.x)

[12] Essl F, Latombe G, Lenzner B, Pagad S, Seebens H, Smith K, Wilson JRU, Genovesi P. 2020 The Convention on Biological Diversity (CBD)’s Post-2020 target on invasive alien species – what should it include and how should it be monitored? NeoBiota **62**, 99-121. (10.3897/neobiota.62.53972)

[13] Van Der Colff D, Kumschick S, Foden W, Wilson JRU. 2020 Comparing the IUCN's EICAT and Red List to improve assessments of the impact of biological invasions. NeoBiota **62**, 509-523. (10.3897/neobiota.62.52623)

[14] Clarke DA, McGeoch MA. 2023 Invasive alien insects represent a clear but variable threat to biodiversity. Curr. Res. Insect Sci. **4**, 100065. (10.1016/j.cris.2023.100065) 37564301 PMC10410178

[15] Crystal-Ornelas R, Lockwood JL. 2020 The ‘known unknowns’ of invasive species impact measurement. Biol. Invasions **22**, 1513-1525. (10.1007/s10530-020-02200-0)

[16] Vujanović D, Losapio G, Milić S, Milić D. 2022 The Impact of Multiple Species Invasion on Soil and Plant Communities Increases With Invasive Species Co-occurrence. Front. Plant Sci. **13**, 875824. (10.3389/fpls.2022.875824) 35712554 PMC9194948

[17] Latombe G, Lenzner B, Schertler A, Dullinger S, Glaser M, Jarić I, Pauchard A, Wilson JRU, Essl F. 2022 What is valued in conservation? A framework to compare ethical perspectives. NeoBiota **72**, 45-80. (10.3897/neobiota.72.79070)

[18] Blackburn TM et al. 2014 A Unified Classification of Alien Species Based on the Magnitude of their Environmental Impacts. PLoS Biol. **12**, e1001850. (10.1371/journal.pbio.1001850) 24802715 PMC4011680

[19] Hawkins CL et al. 2015 Framework and guidelines for implementing the proposed IUCN Environmental Impact Classification for Alien Taxa (EICAT). Diversity Distrib. **21**, 1360-1363. (10.1111/ddi.12379)

[20] Wilson JRU, Faulkner KT, Rahlao SJ, Richardson DM, Zengeya TA, Wilgen BW. 2018 Indicators for monitoring biological invasions at a national level. J. Appl. Ecol. **55**, 2612-2620. (10.1111/1365-2664.13251)

[21] Jetz W et al. 2019 Essential biodiversity variables for mapping and monitoring species populations. Nat. Ecol. Evol. **3**, 539-551. (10.1038/s41559-019-0826-1) 30858594

[22] Clarke DA et al. 2021 Options for reducing uncertainty in impact classification for alien species. Ecosphere **12**, e03461. (10.1002/ecs2.3461)

[23] Probert AF, Volery L, Kumschick S, Vimercati G, Bacher S. 2020 Understanding uncertainty in the Impact Classification for Alien Taxa (ICAT) assessments. NeoBiota **62**, 387-405. (10.3897/neobiota.62.52010)

[24] Pagad S, Genovesi P, Carnevali L, Schigel D, McGeoch MA. 2018 Introducing the Global Register of Introduced and Invasive Species. Sci. Data **5**, 170202. (10.1038/sdata.2017.202) 29360103 PMC5779068

[25] Pagad S et al. 2022 Country Compendium of the Global Register of Introduced and Invasive Species. Sci. Data **9**, 391. (10.1038/s41597-022-01514-z) 35810161 PMC9271038

[26] Seebens H et al. 2020 A workflow for standardising and integrating alien species distribution data. NeoBiota **59**, 39-59. (10.3897/neobiota.59.53578)

[27] Bomford M. 2008 Risk assessment models for establishment of exotic vertebrates in Australia and New Zealand. Canberra, Australia: Invasive Animals Cooperative Research Centre.

[28] Hayes KR, Barry SC. 2008 Are there any consistent predictors of invasion success? Biol. Invasions **10**, 483-506. (10.1007/s10530-007-9146-5)

[29] Byers JE et al. 2015 Invasion Expansion: Time since introduction best predicts global ranges of marine invaders. Sci. Rep. **5**, 12436. (10.1038/srep12436) 26227803 PMC4521186

[30] Essl F, Dullinger S, Rabitsch W, Hulme PE, Pyšek P, Wilson JRU, Richardson DM. 2015 Delayed biodiversity change: no time to waste. Trends Ecol. Evol. **30**, 375-378. (10.1016/j.tree.2015.05.002) 26028440

[31] Gallien L, Douzet R, Pratte S, Zimmermann NE, Thuiller W. 2012 Invasive species distribution models - how violating the equilibrium assumption can create new insights: Beyond the equilibrium assumption of SDMs. Glob. Ecol. Biogeogr. **21**, 1126-1136. (10.1111/j.1466-8238.2012.00768.x)

[32] Pili AN, Tingley R, Sy EY, Diesmos MLL, Diesmos AC. 2020 Niche shifts and environmental non-equilibrium undermine the usefulness of ecological niche models for invasion risk assessments. Sci. Rep. **10**, 7972. (10.1038/s41598-020-64568-2) 32409706 PMC7224218

[33] Latombe G et al. 2017 A vision for global monitoring of biological invasions. Biol. Conserv. **213**, 295-308. (10.1016/j.biocon.2016.06.013)

[34] Kumschick S, Vimercati G, De Villiers FA, Mokhatla MM, Davies SJ, Thorp CJ, Rebelo AD, Measey GJ. 2017 Impact assessment with different scoring tools: How well do alien amphibian assessments match? NeoBiota **33**, 53-66. (10.3897/neobiota.33.10376)

[35] Seebens H. 2021 Alien Species First Records Database. (10.5281/ZENODO.4632335)

[36] GBIF Secretariat. 2023 GBIF Backbone Taxonomy. (10.15468/39OMEI)

[37] Groom Q et al. 2019 Improving Darwin Core for research and management of alien species. Biodivers. Inf. Sci. Stand. **3**, e38084. (10.3897/biss.3.38084)

[38] McGeoch MA, Spear D, Kleynhans EJ, Marais E. 2012 Uncertainty in invasive alien species listing. Ecol. Appl. **22**, 959-971. (10.2307/23213930) 22645824

[39] GBIF. 2022 Occurrence Download. 772939007491. (10.15468/DL.3C2MMC)

[40] Forti LR, Becker CG, Tacioli L, Pereira VR, Santos ACFA, Oliveira I, Haddad CFB, Toledo LF. 2017 Perspectives on invasive amphibians in Brazil. Plos One **12**, e0184703. (10.1371/journal.pone.0184703) 28938024 PMC5609743

[41] Aikio S, Duncan RP, Hulme PE. 2010 Lag-phases in alien plant invasions: separating the facts from the artefacts. Oikos **119**, 370-378. (10.1111/j.1600-0706.2009.17963.x)

[42] Gaiji S, Chavan V, Ariño AH, Otegui J, Hobern D, Sood R, Robles E. 2013 Content assessment of the primary biodiversity data published through GBIF network: Status, challenges and potentials. Biodiv. Inf. **8**, 94-172. (10.17161/bi.v8i2.4124)

[43] Simberloff D. 2011 How common are invasion-induced ecosystem impacts? Biol. Invasions **13**, 1255-1268. (10.1007/s10530-011-9956-3)

[44] Bacher S et al. 2018 Socio-economic impact classification of alien taxa (SEICAT). Methods Ecol. Evol. **9**, 159-168. (10.1111/2041-210X.12844)

[45] Bellard C, Rysman J-F, Leroy B, Claud C, Mace GM. 2017 A global picture of biological invasion threat on islands. Nat. Ecol. Evol. **1**, 1862-1869. (10.1038/s41559-017-0365-6) 29109470

[46] Leclère D et al. 2020 Bending the curve of terrestrial biodiversity needs an integrated strategy. Nature **585**, 551-556. (10.1038/s41586-020-2705-y) 32908312

[47] Zizka A et al. 2019 CoordinateCleaner: Standardized cleaning of occurrence records from biological collection databases. Methods Ecol. Evol. **10**, 744-751. (10.1111/2041-210X.13152)

[48] Leslie PH. 1966 The Intrinsic Rate of Increase and the Overlap of Successive Generations in a Population of Guillemots (*Uria aalge* Pont.). J. Anim. Ecol. **35**, 291-301. (10.2307/2396)

[49] McGeoch MA et al. 2023 Invasion trends: An interpretable measure of change is needed to support policy targets. Conserv. Lett. **16**, e12981. (10.1111/conl.12981)

[50] Bacher S et al. 2021 IPBES Invasive Alien Species Assessment, data management report for Chapter 4. Impact Evidence Database. (10.5281/ZENODO.5766070)

[51] Henriksen MV et al. 2024 Global indicators of the environmental impacts of invasive alien species and their information adequacy. GitHub repository. (https://github.com/EduardoArle/Impacts) 10.1098/rstb.2023.0323 PMC10999262 38583467

[52] Henriksen MV et al. 2024 Global indicators of the environmental impacts of invasive alien species and their information adequacy. Figshare. (10.6084/m9.figshare.c.7090104) PMC10999262 38583467

